# Coated Betaine Improves Lamb Meat Quality and Flavor by Modulating Rumen Microbial Flora

**DOI:** 10.3390/ani16060970

**Published:** 2026-03-20

**Authors:** Shude Shi, Xiongxiong Li, Shangwu Ma, Yuzhu Sha, Yuling Qu, Shengguo Zhao

**Affiliations:** 1 College of Animal Science and Technology, Gansu Agricultural University, Lanzhou 730070, China; 2 Key Laboratory of Animal Genetics and Breeding on Tibetan Plateau, Ministry of Agriculture and Rural Affairs, Lanzhou Institute of Husbandry and Pharmaceutical Sciences of Chinese Academy of Agricultural Sciences, Lanzhou 730050, China; 3 College of Food Science and Engineering, Gansu Agricultural University, Lanzhou 730070, China

**Keywords:** volatile fatty acids, flavor compounds, microorganisms, rumen fermentation, coated betaine

## Abstract

The sensory quality and flavor of lamb meat, critical to market competitiveness, are influenced by rumen microbial fermentation and dietary management strategies. Coated betaine (CBet), a rumen-protected methyl donor, exerts systemic nutritional regulation in ruminants. This study explored the effects of CBet supplementation on lamb meat quality using 18 Dorset ♂ × Hu sheep ♀ F1 crossbred lambs, randomly assigned to either a control group (basal diet) or a 0.20% CBet-supplemented diet for 60 days (*n* = 9 per group). The results demonstrated that CBet significantly increased ruminal concentrations of total volatile fatty acids (TVFAs), acetic acid, propionic acid, and butyric acid (*p* < 0.05). Additionally, CBet supplementation enhanced muscle redness (a*), crude fat, crude ash, heptadecanoic acid (C17:0), and tricosanoic acid (C23:0) (*p* < 0.05) while decreasing shear force and the concentration of cis-13,16-docosadienoic acid (C22:2) (*p* < 0.05). Furthermore, CBet elevated characteristic flavor compounds (e.g., nonanal) and their relative odor activity values (ROAVs), and decreased undesirable odors (e.g., dodecanal) (*p* < 0.05). As illustrated in the graphical abstract, these improvements were mediated through regulatory effects of CBet on rumen microbiota composition, muscle fatty acids, amino acids, and volatile flavor compounds. Specifically, CBet significantly increased the relative abundances of Firmicutes, Proteobacteria, *Prevotella*, and *Bifidobacterium* in the rumen (*p* < 0.05) and altered the Firmicutes/Bacteroidota ratio. In conclusion, dietary supplementation with 0.20% CBet effectively enhances lamb meat quality and flavor, effects closely associated with changes in the abundance of key ruminal microbial taxa.

## Simple Summary

Taste, flavor, and nutritional value of lamb meat are critical factors influencing consumer preference. Developing safe and effective strategies to enhance these attributes is therefore essential for sustainable livestock production. This study investigated whether dietary supplementation with coated betaine (CBet), a natural feed additive, could improve lamb meat quality. Eighteen lambs were randomly allocated to two groups (*n* = 9 per group) and fed either a basal diet (control) or a basal diet supplemented with 0.20% CBet (treatment group) for 60 days. The results showed that CBet significantly improved meat redness, tenderness, and concentrations of beneficial fats and minerals. Notably, CBet increased desirable flavor notes (e.g., floral and roasted aromas) and reduced undesirable odors. These improvements were closely related to alterations in the ruminal microbial community, which promoted the generation of beneficial metabolites. Collectively, this study provides a safe nutritional strategy to improve lamb meat quality, satisfying consumer demands for tastier, healthier meat and supporting high-quality livestock production.

## 1. Introduction

The distinctive flavor profile and high nutritional value of lamb meat constitute key competitive advantages in the global market [1,2]. With the upgrading of consumer demand, there is an increasing preference for lamb meat with tender texture, rich desirable flavor, and balanced nutritional composition (e.g., higher unsaturated fatty acids and lower off-flavor precursors) [3,4]. This has driven the development of safe and efficient nutritional regulation strategies in ruminant production. Previous studies have demonstrated that lamb flavor development primarily depends on muscle fatty acid composition and the profile of volatile compounds generated through lipid oxidation [5], a process closely associated with dietary composition, rumen fermentation characteristics, and microbial metabolic activity [6,7]. As the primary digestive and metabolic organ in ruminants, the rumen harbors complex microbial communities that ferment dietary nutrients into metabolic products, including volatile fatty acids (VFAs). These metabolites not only provide essential energy substrates for the host but also play a crucial role in regulating intramuscular fat deposition and generating flavor precursor compounds [8,9]. Consequently, precise modulation of the rumen microbiota through optimized nutritional strategies has emerged as an important approach for improving lamb meat quality.

Betaine (trimethylglycine), an important methyl donor, participates in multiple physiological processes, including osmoregulation and lipid metabolism in animals [10,11]. In ruminant production systems, betaine is commonly used as a feed additive to enhance animal performance and health [12]. However, unprotected betaine is rapidly degraded in the rumen, which greatly limits its systemic regulatory effects on hindgut and whole-body metabolism [13]. Coating technology can significantly improve the rumen bypass stability of betaine, thereby enhancing its biological availability [14], and different coating materials (e.g., yeast powder, palm oil, and gelatin) and processes have been shown to affect bypass rates (ranging from 50% to 90%) [15,16,17]. Among these, yeast powder has attracted attention due to its biocompatibility, porous structure for effective encapsulation, and inherent bioactive components that synergistically regulate rumen microbiota, making it a promising coating carrier for betaine [18,19]. Previous studies have reported that dietary betaine supplementation improves nutrient digestibility, rumen fermentation efficiency, and microbial diversity in ruminants [20]. Additionally, it can optimize body fat distribution and alter muscle fatty acid composition, suggesting potential benefits for meat quality improvement [21]. Nevertheless, existing studies mainly focus on production performance and selected meat quality traits, and the mechanisms by which coated betaine (CBet) regulates the formation of characteristic lamb flavor compounds through modulation of rumen microbial communities and fermentation patterns remain unclear. Specifically, the regulatory cascade linking CBet, rumen microbiota shift, fermentation metabolite changes, muscle fatty acid remodeling, and volatile flavor compound formation has not been systematically elucidated.

Therefore, this study used weaned Dorset ♂ × Hu sheep ♀ F1 crossbred lambs as the experimental model to systematically investigate the effects of dietary coated betaine (CBet) supplementation on lamb meat quality traits (e.g., meat color, tenderness, and nutritional composition), muscle fatty acid profile (including OBCFAs and key unsaturated fatty acids), and key volatile flavor compounds (e.g., aldehydes, alcohols, and hydrocarbons). In addition, the regulatory effects of CBet on ruminal fermentation parameters (including VFA composition) and rumen bacterial community structure were comprehensively analyzed. Through integrated correlation analyses, this study aims to elucidate the intrinsic relationships within the “rumen microbiota–fermentation metabolism–muscle flavor compound” axis. The findings of this study provide scientific evidence for the application of yeast powder-coated betaine in ruminant production and practical guidance for developing ruminant-specific functional feed additives aimed at precisely improving meat flavor quality.

## 2. Materials and Methods

### 2.1. CBet

The CBet used in this study was provided by Chongqing Ubio Biotechnology Co., Ltd. (Chongqing, China). The product contains 89.0% analytical-grade betaine, with yeast powder as the coating carrier. Yeast powder was chosen due to three key advantages for ruminant feed coating technology: (1) excellent biocompatibility and degradability in the ruminant digestive tract, preventing potential adverse health effects; (2) porous structural characteristics enabling effective encapsulation of betaine, forming a stable physical barrier that resists ruminal microbial degradation; and (3) bioactive components (e.g., mannan-oligosaccharides, β-glucans) that synergistically modulate rumen microbiota and fermentation, aligning with the nutritional goals of this study. The rumen-bypass rate of 70% is based on technical specifications provided by the manufacturer (Chongqing Ubio Biotechnology Co., Ltd.) and was not independently verified in vivo or in situ in this study.

### 2.2. Animals, Diets, and Experimental Design

The animals selected for this study were weaned lambs from a standardized large-scale farm located in Linxia City, Gansu Province, China. All experimental procedures were approved by the Animal Ethics Committee of Gansu Agricultural University (Approval ID: GSAU-2th-AST-2023-053). Eighteen healthy F1 crossbred male lambs (Dorset ♂ × Hu sheep ♀) with an initial mean body weight of 21.58 ± 0.60 kg were randomly assigned into two groups (*n* = 9 per group): a control group (CON) receiving only the basal diet and a CBet-supplemented group receiving the basal diet containing 0.20% CBet. Each group comprised 3 replicates, with 3 lambs per replicate.

CBet (0.20% inclusion rate) was calculated based on the recommendation of its manufacturer (Chongqing Ubio Biotechnology Co., Ltd.) and relevant previous studies [12,21]. The basal diet was formulated into total mixed ration (TMR) pellets following the Feeding Standard of Meat Sheep (Ministry of Agriculture, China; Table S1). According to communication with Chongqing Ubio Biotechnology Co., Ltd., the coated betaine can withstand the temperature during the high-temperature pelleting process and maximize the retention of coating integrity. Lambs had ad libitum access to TMR and fresh drinking water, with feeding conducted twice daily (08:00 and 18:00). During feed production, 0.20% CBet was homogeneously mixed into the basal diet to form TMR pellets. The experimental design consisted of an adaptation period of 10 days followed by a 60-day treatment period. Before the trial began, pens were thoroughly disinfected, and lambs were dewormed and ear-tagged. At the end of the experiment, 6 lambs from each group were randomly selected and slaughtered according to the Operating Procedures on Sheep and Goat Slaughter (NY/T 3469-2019 [22], Ministry of Agriculture, China).

### 2.3. Sample Collection and Processing

Following slaughter, *Longissimus dorsi* (LD) muscle samples were excised from the left carcass between the 12th and 13th ribs. A portion of each sample was immediately analyzed onsite for muscle pH, cooking loss, and drip loss, while the remainder was stored at −20 °C for subsequent analyses of muscle fatty acid composition and volatile flavor compounds. Simultaneously, rumen fluid samples were filtered through four layers of sterile gauze into precooled cryogenic vials, rapidly frozen in liquid nitrogen, transported under low-temperature conditions, and stored at −80 °C for subsequent analyses of ruminal fermentation parameters and 16S rRNA gene sequencing.

### 2.4. Meat Quality Analysis

#### 2.4.1. Analysis of Muscle Physicochemical Indices and Routine Nutrients

Meat color parameters (L* = lightness, a* = red index, b* = yellow index) were determined according to the CIELAB scale (Commission Internationale de l’Éclairage [CIE], 1976 [23]), and pH values were determined using a pH meter (Testo 205, Testo, Titisee-Neustadt, Germany). Intramuscular fat content was determined according to the Official Methods of Analysis of AOAC International (Method 920.39C: Crude Fat in Meat and Meat Products) [24]. *Longissimus lumborum* samples were sealed in polyethylene bags and heated in a water bath until the internal temperature reached 70 °C, as monitored using a digital thermometer. The samples were then cooled to 35 °C, and meat tenderness was determined using a digital display muscle tenderness meter (C-LM3B, College of Engineering, Northeast Agricultural University, China). Cooking loss was also determined by heating the samples in an 85 °C water bath for 30 min [25]. For drip loss determination, meat samples were cut perpendicular to the direction of muscle fibers, weighed (W1), stored at 4 °C for 24 h in plastic containers, and then reweighed (W2). Drip loss (%) was calculated using the following formula: Drip Loss (%) = [(W1 − W2)/W1] × 100. Moisture, ash, crude protein (CP), and ether extract (EE) contents in muscle were determined according to the method described by Cantarero [26]. These meat quality indicators and fatty acid profiles are widely used to evaluate the effects of nutritional strategies on goat and kid meat quality [27].

#### 2.4.2. Analysis of Fatty Acid Composition

According to the method described by Wang et al. [28], *Longissimus dorsi* (LD) muscle samples were thawed and homogenized thoroughly. A 1.0 g aliquot was accurately weighed into a 10 mL screw-cap tube, followed by the addition of 0.7 mL of 10 mol/L potassium hydroxide (KOH) solution and 5.3 mL of anhydrous methanol. The tubes were incubated in a 55 °C water bath for 1.5 h with intermittent vortex mixing every 5 s and then cooled to room temperature (≤25 °C) using running tap water. Subsequently, 0.58 mL of 12 mol/L sulfuric acid (H_2_SO_4_) solution was added, and the mixture was subjected again to water bath incubation and vortex mixing under the same conditions. The reaction mixture was then mixed with 3 mL of n-hexane and vortexed, after which the upper organic phase was transferred to centrifuge tubes. The samples were centrifuged at 3000× *g* for 5 min, and the supernatant was filtered through an organic-phase membrane into a sample vial and concentrated to approximately 1.5 mL at 45 °C. Fatty acid composition was determined using a gas chromatograph (GC-2010 Plus, Shimadzu, Kyoto, Japan) equipped with an SPTM™-2560 capillary column (100 m × 0.25 mm × 0.20 μm). Chromatographic conditions were set as follows: injector temperature 250 °C; carrier gas nitrogen at a flow rate of 1.0 mL/min; injection volume 1.0 μL (splitless mode). Relative percentages of individual fatty acids were calculated using the peak area normalization method, and the mean relative percentage of each fatty acid was used for statistical analysis.

#### 2.4.3. Analysis of Meat Volatile Flavor Compounds

According to the procedure by Wang et al. [29], 5.0 g of homogenized lamb *Longissimus dorsi* (LD) muscle was placed into a 10 mL headspace vial, followed by the addition of 2 g high-purity sodium chloride (NaCl) and 1 μL of 0.1632 μg/μL 2-methyl-3-heptanone (internal standard). After sealing and vigorous vortex mixing, the vials were incubated in a thermostatic water bath at 90 °C for 1 h. This extraction condition was selected based on several considerations: (1) Heating at 90 °C for 1 h is widely used in studies of meat volatile flavor compounds and effectively releases low-volatility flavor components such as aldehydes and heterocyclic compounds. Meanwhile, the natural precursor compounds in meat regulate Maillard reactions, and both lipid oxidation and Maillard reaction rates remain moderate under this temperature condition, preventing excessive reactions [30,31,32]. (2) The condition simulates the mild cooking process of lamb meat (e.g., braising or stewing), making the detected flavor profile more representative of consumer sensory perception. (3) The addition of NaCl promotes the partitioning of volatile compounds into the headspace through a salting-out effect and suppresses endogenous oxidase activity in muscle tissue, thereby reducing the risk of de novo lipid oxidation during extraction [33].

A preconditioned solid-phase microextraction (SPME) fiber (50/30 μm DVB/CAR/PDMS) was inserted into the headspace of the sealed vial using a syringe holder and exposed for 40 min to adsorb volatile compounds. The fiber was then retracted and immediately desorbed in the injection port of a gas chromatography–mass spectrometry (GC-MS) system at 250 °C for 5 min prior to analysis. GC-MS conditions were as follows: injection port temperature 250 °C; carrier gas high-purity helium; oven temperature program starting at 50 °C with a 1 min hold, followed by heating at 3.5 °C/min to 220 °C and holding at 220 °C for 20 min. Compound identification was performed by comparing mass spectra with the National Institute of Standards and Technology (NIST) database (~107,000 compounds) and the Wiley Registry database (v6.0; ~320,000 compounds). Only compounds with a match factor ≥800 and spectral purity ≥800 were accepted for identification. Relative contents of volatile compounds were determined using the peak area normalization method.

The relative odor activity value (ROAV) was calculated using the following equation: ROAV = 100 × (*C*_i_/*C*_max_) × (*T*_max_/*T*_i_). In this equation, *C*_i_ and *T*_i_ represent the relative concentration (μg/kg) and odor threshold of the target volatile compound, respectively, whereas *C*_max_ and *T*_max_ represent the concentration and odor threshold of the compound contributing most strongly to flavor. The compound with the greatest flavor contribution is assigned an ROAV value of 100. Compounds with ROAV ≥ 1 are considered key aroma-active compounds, whereas those with ROAV < 0.1 are regarded as minor contributors that slightly modify the overall flavor profile [34].

### 2.5. Rumen Fermentation Characteristics

Ruminal fluid pH was immediately measured using a pH meter (Model P611; Leici Instrument Co., Ltd., Shanghai, China) equipped with a glass electrode after sample collection and filtration. Ammonia nitrogen (NH_3_-N) concentrations were determined using the colorimetric method on a TU-1901 ultraviolet–visible spectrophotometer (Persee General Instrument Co., Ltd., Beijing, China), as previously described [35]. VFAs in ruminal fluid were quantified by gas chromatography (GC-2010 Plus; Shimadzu Corporation, Kyoto, Japan) equipped with a flame ionization detector (FID).

### 2.6. DNA Extraction and 16s rRNA Sequencing

Genomic DNA was extracted from ruminal fluid samples using the MN NucleoSpin 96 Soil kit (Macherey-Nagel, Düren, Germany), and DNA concentration and purity were assessed with a NanoDrop 2000 UV-Vis spectrophotometer (Thermo Fisher Scientific, Wilmington, DE, USA). The V3-V4 hypervariable regions of bacterial 16S rRNA genes were amplified using primers 338F (5′-ACTCCTACGGGAGGCAGCA-3′) and 806R (5′-AGGACTACHVGGGTWTCTAAT-3′). PCR products were purified using the Omega DNA Cleanup Kit (Omega Bio-tek, Norcross, GA, USA) and subsequently subjected to paired-end high-throughput sequencing (2 × 250 bp) on an Illumina NovaSeq 6000 sequencing platform (Illumina, Inc., San Diego, CA, USA). Before bioinformatic analysis, raw sequence data underwent quality control and filtering. Bioinformatic processing included sequence merging using FLASH v1.2.7, quality trimming with Trimmomatic v0.33, chimera removal using UCHIME v4.2, and OTU clustering at 97% sequence similarity with Usearch v11.0. Taxonomic classification of representative OTU sequences was performed using the SILVA 138 database [36].

### 2.7. Statistical Analysis

Experimental data were compiled using Microsoft Excel 2019 and statistically analyzed using IBM SPSS Statistics 22.0. Results are expressed as means ± standard error of the mean (SEM). Intergroup differences in meat quality, fatty acids, volatile flavor compounds, rumen fermentation parameters, and microbial genus abundances were compared using an independent-samples *t*-test, with significance set at *p* < 0.05 and high significance at *p* < 0.01. For microbial diversity analyses, α-diversity indices were tested using *t*-tests via QIIME2, while β-diversity was evaluated by principal coordinate analysis (PCoA) coupled with ANOSIM tests. Linear discriminant analysis effect size (LEfSe; LDA score > 2.5) was utilized to identify significantly different microbial taxa, and genus-level validation was performed by Metastats using *t*-tests. Spearman rank correlation analysis was conducted to examine correlations among rumen fermentation parameters, fatty acids, flavor compounds, and the top 20 microbial genera, with significance determined at *p* < 0.05.

## 3. Results

### 3.1. Muscle Physicochemical Indices and Routine Nutrients

**Figure 1:**
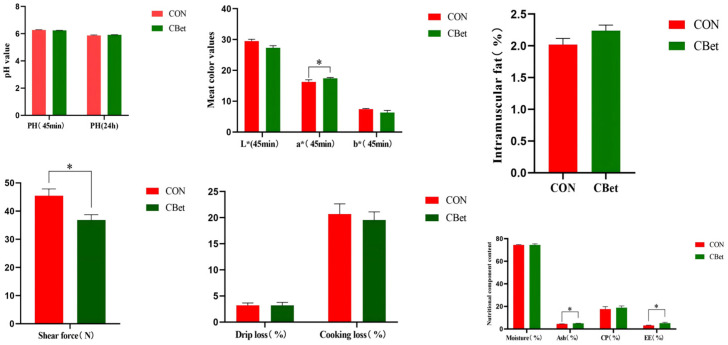
Effects of dietary CBet supplementation on physicochemical indices and general nutritional components in lamb muscle. Values are expressed as means ± SEM (*n* = 6); * Indicates significant difference between the two groups (*p* < 0.05).

As presented in Figure 1, dietary supplementation with CBet significantly increased muscle redness (a* value), crude fat (ether extract, EE), and crude ash contents (*p* < 0.05) while significantly reducing muscle shear force (*p* < 0.05). Although no significant differences in yellowness (b* value) and cooking loss were observed between the groups (*p* > 0.05), both parameters showed numerically lower values in the CBet group compared to the control (CON group).

### 3.2. Fatty Acids Composition

A total of 35 fatty acids were identified in lamb muscle samples, comprising 15 saturated fatty acids (SFAs), 9 monounsaturated fatty acids (MUFAs), and 11 polyunsaturated fatty acids (PUFAs) (Table 1). Dietary CBet supplementation altered the muscle fatty acid profile. Specifically, it significantly increased the concentrations of heptadecanoic acid (C17:0) and tricosanoic acid (C23:0) among SFAs (*p* < 0.05) while significantly decreasing the concentration of cis-13,16-docosadienoic acid (C22:2) among PUFAs (*p* < 0.05). Compared with the control (CON) group, lambs receiving CBet supplementation exhibited increasing trends in total muscle unsaturated fatty acids (UFAs), MUFAs, and PUFAs, alongside a decreasing trend in total SFAs, although these differences did not reach statistical significance (*p* > 0.05).

### 3.3. Volatile Compound Composition

**Figure 2:**
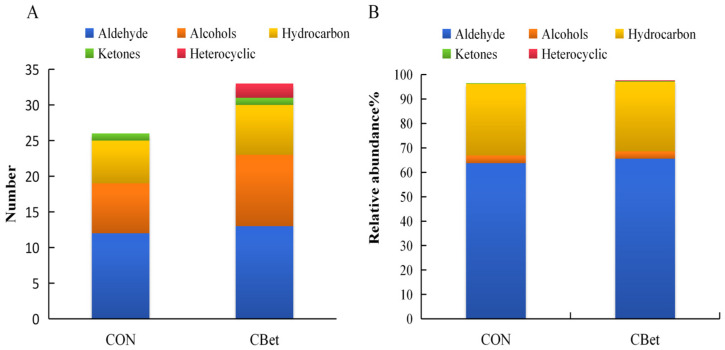
Numbers (**A**) and relative contents (**B**) of volatile flavor compounds in the two groups.

A total of 26 and 33 volatile compounds were identified in the CON and CBet groups, respectively. These compounds were classified into five categories: aldehydes, alcohols, ketones, hydrocarbons, and heterocyclic compounds (Figure 2). Interestingly, seven additional volatile compounds were detected exclusively in the CBet group. Comparisons of volatile compound contents (Table 2) showed that dietary supplementation with CBet significantly increased the levels of nonanal, benzaldehyde, and 1-octen-3-ol in lamb muscle (*p* < 0.05), whereas levels of dodecanal, (E)-2-decenal, 1-nonanol, cycloheptanol, ethylbenzene, p-xylene, and o-xylene were significantly reduced (*p* < 0.05). Contents of the remaining volatile compounds showed no statistically significant differences (*p >* 0.05).

To further elucidate the contributions of volatile compounds to lamb flavor, their relative odor activity values (ROAVs) were calculated (Table 3). Results indicated that (E,E)-2,4-nonadienal made the greatest contribution to lamb flavor in both groups, assigned an ROAV of 100 (the highest flavor-impact compound by definition). Using an ROAV threshold of >0.01, a total of 21 and 23 key flavor compounds were identified in the CON and CBet groups, respectively. Comparative analysis revealed that nonanal and 1-octen-3-ol exhibited higher ROAVs in the CBet group compared to the CON group. Conversely, hexanal, heptanal, octanal, (E)-2-decenal, and (E,E)-2,4-decadienal displayed lower ROAV values in the CBet group.

### 3.4. VFAs

**Figure 3:**
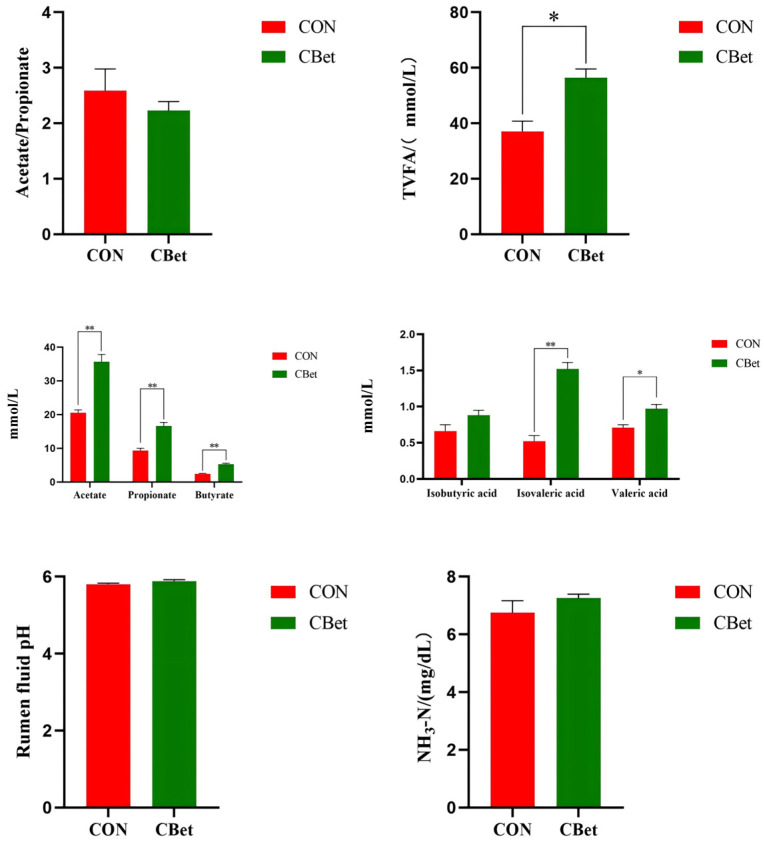
Effects of dietary CBet supplementation on rumen fermentation parameters of F1 lambs in Tao Lake. Values are expressed as means ± SEM (*n* = 6); * indicates significant differences (*p* < 0.05), ** indicates highly significant differences (*p* < 0.01).

As shown in Figure 3, dietary supplementation with CBet significantly increased the concentrations of valeric acid and total volatile fatty acids (TVFA) in ruminal fluid (*p* < 0.05). Additionally, highly significant increases were observed for concentrations of acetate, propionate, butyrate, and isovaleric acid (*p* < 0.01). However, supplementation with CBet had no significant effect on ruminal pH or ammonia nitrogen (NH_3_-N) concentrations (*p* > 0.05).

### 3.5. Rumen Bacterial Microbiota

**Figure 4:**
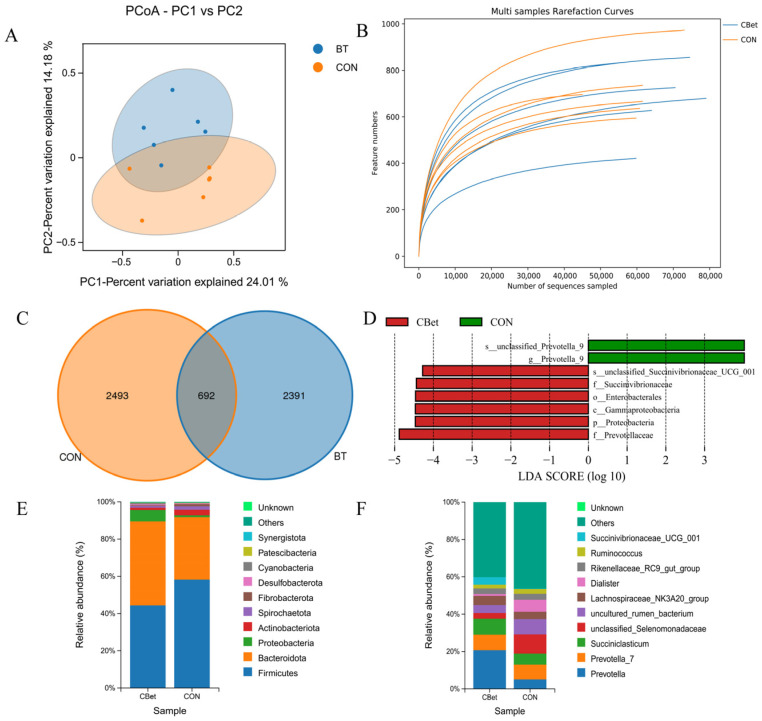
Effects of dietary CBet supplementation on rumen microbial composition. (**A**) Principal coordinate analysis (PCoA); (**B**) OTU rarefaction curves; (**C**) OTU-based Venn diagram; (**D**) differential bacterial taxa identified by linear discriminant analysis effect size (LEfSe); (**E**) relative abundance at the phylum level; (**F**) relative abundance at the genus level.

Raw sequencing yielded 987,232 reads, and after quality control and trimming, 848,124 high-quality reads remained, averaging 70,677 reads per sample. Principal coordinates analysis (PCoA) based on weighted UniFrac distances revealed differences in ruminal bacterial community structure between groups, although the separation was incomplete (R^2^ = 0.124, *p* = 0.085). Principal coordinates 1 and 2 explained 24.01% and 14.18% of total variance, respectively (Figure 4A). Dietary CBet supplementation did not significantly affect microbial α-diversity (Shannon and Simpson indices) or species richness (ACE and Chao indices) (*p* > 0.05, Table S2). OTU rarefaction curves indicated sufficient sequencing depth for subsequent analyses (Figure 4B). Venn diagram analysis identified 692 shared OTUs, with 2391 and 2483 unique OTUs detected in the CBet and CON groups, respectively (Figure 4C).

At the phylum level, Firmicutes, Bacteroidota, and Proteobacteria were the predominant bacterial groups in lamb rumen (Figure 4E). Microbial taxa analysis (relative abundance > 0.1%) indicated that dietary CBet supplementation significantly increased the relative abundances of Firmicutes and Proteobacteria and decreased Bacteroidota compared to the CON group (*p* < 0.05). At the genus level, *Prevotella* and *Succiniclasticum* showed significantly higher relative abundances in the CBet group (*p* < 0.05), whereas no significant differences were observed among other detected genera. LEfSe analysis (Figure 4F) identified *Prevotellaceae*, Proteobacteria, and Gammaproteobacteria as taxa significantly enriched in the CBet group, while *Prevotella* was the dominant genus in the CON group (Figure 4D).

### 3.6. Correlation Analysis

**Figure 5:**
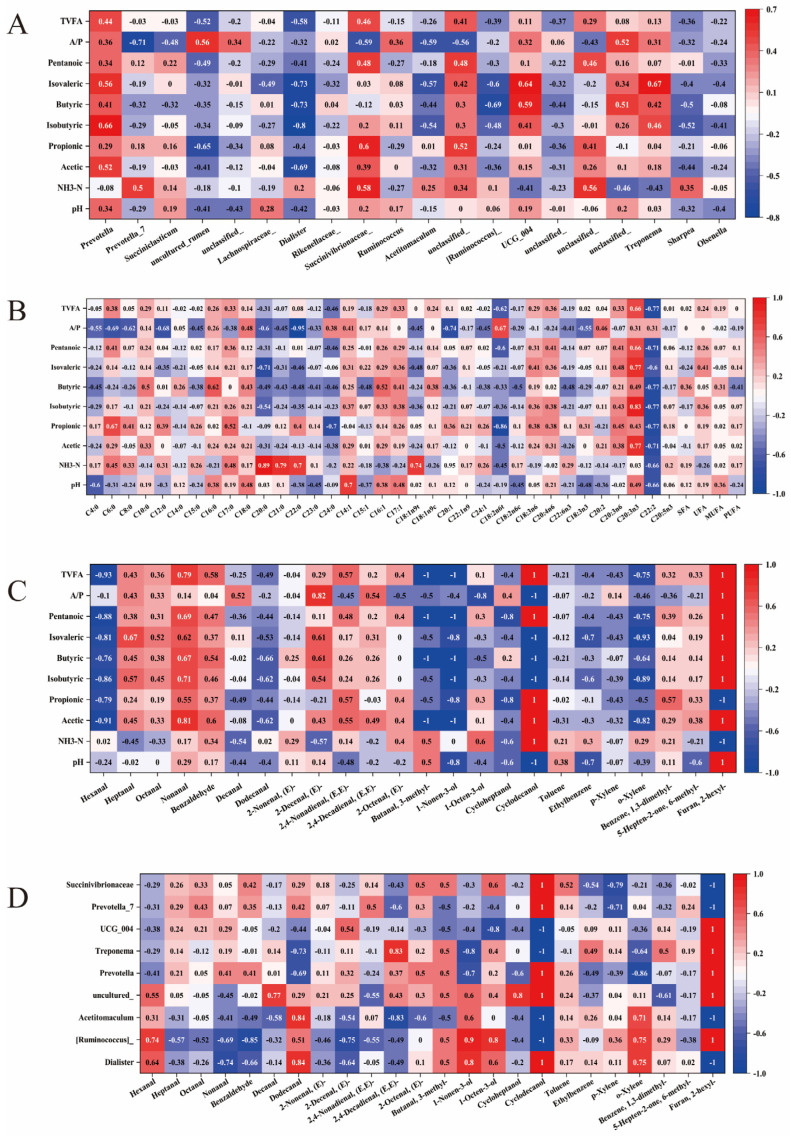
Correlation analysis. (**A**) Correlation analysis between rumen fermentation parameters and the top 20 microbial genera; (**B**) correlation analysis between rumen fermentation parameters and muscle fatty acids; (**C**) correlation analysis between rumen fermentation parameters and flavor compounds; (**D**) correlation analysis between the top 20 differentially expressed microbial genera and flavor compounds. Values represent correlation coefficients; red indicates positive correlations, and blue indicates negative correlations.

Correlation analysis between the top 20 rumen microbial genera and rumen fermentation parameters (Figure 5A) indicated that *Dialister* exhibited significant negative correlations with butyric acid, isobutyric acid, isovaleric acid, acetic acid, and TVFA (*p* < 0.05). [*Ruminococcus*]_gauvreauii_group showed significant negative correlations with butyric acid and isovaleric acid (*p* < 0.05), while *Acetitomaculum* negatively correlated with the acetate-to-propionate ratio (A/P), and Uncultured_rumen_bacterium negatively correlated with propionic acid. Additionally, *Prevotella* exhibited a significant positive correlation with isobutyric acid (*p* < 0.05), *Treponema* positively correlated with isovaleric acid, and *UCG_004* positively correlated with butyric acid and isovaleric acid. Correlation analysis between rumen fermentation parameters and muscle fatty acid composition (Figure 5B) demonstrated positive correlations between NH_3_-N and C20:0, C21:0, C18:1n9t, and C20:1; a negative correlation between propionic acid and C18:2n6t; a positive correlation between isobutyric acid and C20:3n3; and a negative correlation between the A/P ratio and C22:0. Correlations between rumen fermentation parameters and key volatile flavor compounds (Figure 5C) indicated negative correlations between hexanal, nonanal, (E)-2-decenal, and 3-methyl-butanal and fermentation parameters (acetic acid, butyric acid, pentanoic acid, and TVFA, respectively). 1-nonen-3-ol and o-xylene negatively correlated with acetic acid, isobutyric acid, butyric acid, pentanoic acid, and TVFA.

Additionally, significant correlations between the top 20 differential microbial genera and primary muscle flavor compounds (Figure 5D) revealed that hexanal positively correlated with [*Ruminococcus*]_gauvreauii_group (*p* < 0.05), nonanal negatively correlated with Dialister (*p* < 0.05), (E,E)-2,4-decadienal negatively correlated with *Acetitomaculum* but positively correlated with *Treponema* (*p* < 0.05), 1-nonen-3-ol positively correlated with [*Ruminococcus*]_gauvreauii_group (*p* < 0.05), p-xylene negatively correlated with Succinivibrionaceae_UCG_001 (*p* < 0.05), and o-xylene negatively correlated with *Prevotella* (*p* < 0.05).

## 4. Discussion

This study demonstrated that dietary supplementation with 0.20% CBet systematically modulates metabolic pathways from rumen fermentation to muscle tissue, effectively enhancing lamb meat quality in terms of palatability, nutritional value, and flavor attributes. The unique regulatory effects of CBet derive from its 70% rumen bypass rate, achieved through yeast powder-based coating technology. This feature allows simultaneous modulation of rumen fermentation and hindgut absorption, fundamentally distinguishing it from uncoated betaine. This research thus provides both practical and theoretical foundations for nutritional strategies to optimize meat quality in weaned lambs.

### 4.1. CBet Improves Physicochemical and Nutritional Qualities of Lamb Meat

The sensory attributes of lamb meat significantly influence consumer preference and purchase decisions, directly affecting market acceptance [37]. Studies by Dong et al. [21] reported that dietary CBet supplementation significantly increased muscle redness (a* value), reduced shear force, and decreased drip loss, aligning well with findings from the present study and further confirming the efficacy and reliability of CBet in enhancing lamb meat sensory quality. Mechanistically, increased muscle redness is closely associated with higher myoglobin content and improved myoglobin structural stability. Reduced shear force and drip loss indicate optimized meat tenderness, primarily due to the improved myofibrillar structure and enhanced cellular water-holding capacity resulting from CBet-mediated regulation of muscle cellular physiological metabolism [38].

In addition to improving sensory attributes, another significant observation from this study was that lamb muscle crude fat (ether extract, EE) and crude ash (Ash) contents were significantly higher in the CBet group, demonstrating the ability of CBet to enhance the nutritional value of lamb meat. The increase in muscle crude fat likely reflects CBet’s effectiveness as a nutritional factor that promotes intramuscular fat deposition by regulating lipid metabolism pathways [39]. Liu et al. [40] confirmed that appropriate nutritional interventions can influence intramuscular fat deposition through modulation of lipid metabolism, simultaneously enhancing meat tenderness and color; this provides strong mechanistic support for the current findings. The significant increase in muscle ash content observed in the CBet group may be associated with betaine’s role as an effective methyl donor, involved in mineral metabolism at the cellular level and thereby promoting mineral deposition within muscle tissue. Notably, such an effect has not been documented in comparable studies, suggesting a promising direction for future research into the mechanisms underlying CBet’s influence on mineral metabolism in ruminants.

### 4.2. CBet Modulates Muscle Fatty Acid Composition to Optimize Flavor Precursors

Fatty acid composition is a critical determinant of lamb meat nutritional value and flavor quality [41]. In this study, dietary supplementation with CBet resulted in increasing trends in total monounsaturated fatty acids (MUFAs) and polyunsaturated fatty acids (PUFAs), accompanied by a decreasing trend in total saturated fatty acids (SFAs). Although these changes did not reach statistical significance (*p* > 0.05), they align with findings by Jin et al. [42] suggesting that betaine can regulate fat deposition in ruminants. However, specific differences in fatty acid profiles observed here highlight the unique advantage of CBet in modulating ruminant lipid metabolism. These unique effects can be primarily attributed to the high rumen-bypass rate (70%) conferred by the yeast-based coating technology, forming a physical barrier that resists microbial degradation within the rumen, thus fundamentally altering the absorption and utilization pathways of betaine. Unlike uncoated betaine, which is rapidly degraded and exhibits limited local effects in the rumen [13], the protected CBet bypasses the rumen effectively and is absorbed predominantly in the hindgut (ileum and colon). Subsequently, it enters systemic circulation, directly participating in muscle methyl metabolism and fatty acid synthesis. This systemic regulatory mechanism of CBet underlies the significant alterations observed in specific muscle fatty acids, such as the notable increase in heptadecanoic acid (C17:0) and the significant decrease in cis-13,16-docosadienoic acid (C22:2). In addition, the rumen-bypass characteristic of CBet reduces its excessive consumption within the rumen, ensuring adequate availability of methyl donors for muscular fat deposition and mineral metabolism. This mechanism likely accounts for the significant elevation of muscle crude fat and crude ash contents in lambs receiving CBet supplementation. Notably, the concentration of margaric acid (C17:0) was significantly elevated in the CBet group. As a characteristic rumen fermentation product, the accumulation of C17:0 generally indicates enhanced activity of fiber-degrading rumen bacteria and the increased production of fatty acid precursors such as propionic acid [43]. Therefore, CBet supplementation may indirectly influence de novo muscle fatty acid synthesis by modulating rumen microbial fermentation patterns. Conversely, the observed significant decrease (*p* < 0.05) in cis-13,16-docosadienoic acid (C22:2) content following CBet supplementation is particularly important, given that this fatty acid is associated with undesirable greasy off-flavors in lamb meat. Thus, its reduction directly contributes to improved flavor quality, highlighting CBet’s targeted efficacy in fatty acid metabolism regulation.

In terms of flavor optimization, the contents of straight-chain saturated fatty acids, including butyric acid (C4:0), capric acid (C10:0), palmitic acid (C16:0), and stearic acid (C18:0), showed slight decreases in the CBet group compared with the CON group, though these differences were not statistically significant (Table 1). Such straight-chain saturated fatty acids typically have limited direct effects on the characteristic “muttony” flavor of lamb meat. The distinctive mutton-like aroma in lamb meat primarily originates from branched-chain fatty acids (BCFAs), notably 4-methyloctanoic acid and 4-methylnonanoic acid, synthesized by rumen microbial metabolism [44,45]. The observed reduction in straight-chain saturated fatty acids in the CBet group, combined with the significant decline in flavor-negative cis-13,16-docosadienoic acid (C22:2), suggests that CBet indirectly influences rumen microbial communities to inhibit synthesis pathways of negative flavor precursors, thereby enhancing lamb meat flavor acceptability for consumers. From a nutritional and health-promoting perspective, it is widely recognized that excessive dietary saturated fatty acid (SFA) intake increases the risk of cardiovascular disease in humans [46], whereas unsaturated fatty acids confer protective effects against cardio-cerebrovascular diseases [47]. In this study, although the changing trends in total muscle SFA, MUFA and PUFA did not reach statistical significance (*p* > 0.05), their directional trends still highlight the potential of CBet to optimize lamb muscle fatty acid profiles. Consequently, CBet supplementation not only holds promise for improving lamb meat flavor through adjustments in fatty acid proportions but also for enhancing the nutritional and health-promoting attributes of lamb meat, ultimately improving its comprehensive market appeal.

### 4.3. CBet Regulates Volatile Flavor Compounds to Enhance Lamb Meat Flavor

Flavor represents a crucial factor influencing the taste and consumer acceptance of lamb meat, with primary volatile flavor components including aldehydes, alcohols, ketones, hydrocarbons, and heterocyclic compounds [48]. The volatile compound profiles identified in this study reflect both the intrinsic characteristics of raw lamb meat and the flavors produced during mild cooking conditions (simulating actual consumption). Thus, the findings have significant practical relevance to lamb meat consumption scenarios. Aldehydes, primarily arising from lipid peroxidation, typically possess low odor thresholds and high aroma intensity, contributing notably to fruity and floral flavors and constituting major contributors to lamb meat aroma [49]. Aldehydes represented the highest relative proportion among all detected volatile compounds in both experimental groups. Key aldehydes (ROAV > 1) identified included nonanal, hexanal, heptanal, (E,E)-2,4-decadienal, octanal, and (E)-2-decenal. Notably, nonanal, characterized by typical floral and roasted notes, is derived from unsaturated fatty acid degradation in lamb meat. Its aldehyde functional group binds specifically to human olfactory receptors, thereby eliciting characteristic aromatic sensations [50]. Although hexanal imparts fresh grassy and herbal odors at lower concentrations, it can lead to rancid and off-flavors when accumulated excessively. In this study, the lower ROAV value of hexanal observed in the CBet group compared to the CON group is beneficial for improving lamb meat palatability. Additionally, benzaldehyde content was significantly elevated in the CBet group. Benzaldehyde (ROAV > 0.1), imparting distinctive almond-like and caramelized sweet notes, is produced via oxidative degradation of phenylalanine or linolenic acid [51]. Therefore, it is plausible that CBet promotes benzaldehyde formation by modulating these oxidative degradation pathways, thus enhancing the overall lamb meat flavor profile.

Additionally, dodecanal is regarded as an undesirable aroma compound in ruminant meat; its excessive accumulation leads to unpleasant fatty and pungent odors, negatively impacting consumer acceptance. This undesirable effect results primarily from its high affinity for lipid substrates and accumulation during improper lipid metabolism, deteriorating meat flavor quality [52,53]. The significant reduction in dodecanal content in the CBet group (*p* < 0.05) confirms the targeted regulatory efficacy of CBet in optimizing lipid metabolism and minimizing the production of undesirable aldehydes, ultimately improving lamb meat flavor.

Alcohol compounds in lamb meat typically originate from fatty acid derivatives, lipid oxidation, or carbonyl reduction processes, imparting distinctive flavors to meat [54]. In this study, the content and ROAV of 1-octen-3-ol significantly increased in the CBet group. This compound, characterized by mushroom-like notes, significantly contributes to desirable aromas in lamb meat. Conversely, concentrations of 1-nonanol and cycloheptanol significantly decreased in the CBet group. Elevated levels of these two alcohols typically induce unpleasant alcoholic or woody odors; thus, their reduced levels are favorable for enhancing lamb meat flavor quality. Previous literature indicates that alcohols generally result from cleavage and subsequent oxidation of unsaturated fatty acids, particularly linoleic acid [34]. This is consistent with the observed relative abundance of linoleic acid and other unsaturated fatty acids in the CBet group, further validating the metabolic effects of CBet observed in this study.

Hydrocarbon compounds typically possess higher flavor thresholds, thus contributing minimally to the overall lamb meat flavor profile. Moreover, some hydrocarbons impart unpleasant solvent-like or plastic-like aromas, making their reduction beneficial for lamb meat flavor improvement [55]. The primary hydrocarbons identified in both groups (ROAV > 0.1) were toluene, ethylbenzene, o-xylene, p-xylene, and 1,3-dimethylbenzene.

In the CBet group, the relative contents of ethylbenzene, p-xylene, and o-xylene significantly decreased. These three aromatic hydrocarbons generally exhibit faint solvent-like odors, and their reduced abundance can effectively minimize undesirable off-flavors, enhancing lamb meat flavor quality [56].

### 4.4. CBet Modulates Rumen Fermentation and Microbial Community as the Core Mechanism

Dynamic shifts in rumen fermentation parameters and microbial community structure constitute the central mechanism by which nutritional interventions influence lamb meat quality [57]. In this current study, dietary supplementation with CBet significantly increased the concentrations of ruminal VFAs, including acetic acid, propionic acid, butyric acid, and TVFA. These findings align with the results of Liu et al. [58], who demonstrated that betaine supplementation enhanced rumen fermentation and microbial community composition, further supporting the beneficial regulatory effects of CBet on rumen metabolism in lambs.

At the genus level, the relative abundance of *Prevotella* significantly increased in the CBet group. As a core genus involved in carbohydrate and protein degradation in the rumen [59], increased abundance of *Prevotella* promotes the fermentation of dietary substrates, leading to enhanced acid production and providing a microbial basis for elevated ruminal VFA levels. Additionally, the relative abundance of *Succiniclasticum*, a succinate-utilizing genus, simultaneously increased. This genus converts succinic acid to propionic acid [60], directly contributing to the observed increase in propionic acid concentration and the slight decrease in the acetate-to-propionate (A/P) ratio in the CBet group.

At the phylum level, the ratio of Firmicutes to Bacteroidota (F/B ratio) significantly increased in the CBet group, consistent with findings reported by Zhu et al. [61]. An elevated ruminal F/B ratio enables lambs to more efficiently utilize dietary energy, promoting energy storage and fat deposition. Thus, CBet supplementation potentially enhances dietary energy conversion efficiency and fat deposition by modulating rumen microbial composition at the phylum level.

It should be noted that the 70% rumen-bypass rate of CBet reported in this study is based on the manufacturer’s technical specifications. Therefore, the initial regulation of rumen fermentation is driven by approximately 30% rumen-available CBet (equivalent to 0.06% of the dietary inclusion). However, the highly significant elevation (*p* < 0.01) of major rumen VFAs observed here is not solely attributable to the small amount of rumen-available betaine. Rather, it results from a synergistic interaction among the rumen-available betaine, the inherent regulatory properties of the yeast powder coating carrier, and the sustained-release effect conferred by the coating technology. Specifically, the yeast powder used as the coating carrier contains bioactive substances such as mannan-oligosaccharides, nucleotides, and β-glucans [35,62,63], which independently modulate rumen microbial composition by enhancing proliferation of fiber-degrading and acid-producing bacteria (e.g., *Prevotella*, *Succiniclasticum*). This modulation significantly promotes rumen fermentation and VFA production. Moreover, the coating technology not only protects betaine from rapid ruminal degradation but also allows for its gradual release under rumen microbial action and fermentation conditions [21,42]. Consequently, a small quantity of rumen-available betaine is released continuously and steadily rather than abruptly, prolonging its interaction with the ruminal microbiota. This sustained-release mechanism significantly amplifies betaine’s influence on rumen fermentation, leading to markedly increased concentrations of major VFAs. Additionally, the combination of slow-release betaine and yeast powder carrier collectively shapes the rumen microbiota, increasing the Firmicutes-to-Bacteroidota (F/B) ratio and enriching acid-producing genera. This microbial synergy further intensifies the fermentation response, accounting for the substantial shifts observed in rumen fermentation parameters.

### 4.5. Correlation Analysis Reveals the Microbe-Fermentation-Flavor Regulatory Cascade

Correlation analyses further elucidated the regulatory cascade linking rumen microbiota through fermentation metabolites to muscle flavor compounds, validating the core hypothesis of the “rumen microbiota–metabolite–meat quality association” previously proposed by Chang et al. [64]. Specifically, *Prevotella* was positively correlated with isobutyric acid, which itself positively correlated with C20:3n3, ultimately influencing the synthesis of volatile aldehydes through fatty acid metabolism. Conversely, Dialister negatively correlated with butyric acid, which in turn negatively correlated with hexanal, an undesirable flavor compound. Thus, changes in Dialister abundance could indirectly reduce hexanal accumulation through modulation of butyric acid levels. These microbiota-metabolite–flavor compound correlations represent novel findings not previously reported, providing new theoretical evidence supporting CBet’s targeted regulation of lamb meat quality. Furthermore, Succinivibrionaceae_UCG_001 positively correlated with ammonia nitrogen (NH_3_-N) and propionic acid, aligning with prior research on ruminal metabolism in ruminants [65]. This confirms the important role of this taxon in rumen protein degradation and propionate production pathways.

In summary, dietary supplementation with CBet can effectively enhance lamb meat quality, nutritional profile, and flavor characteristics by selectively modulating rumen microbial community structure and optimizing rumen fermentation parameters, thereby regulating fatty acid metabolism in muscle tissue. This study provides robust mechanistic insights into nutritional interventions aimed at improving lamb meat quality via microbial and metabolic modulation.

### 4.6. Study Limitations and Future Perspectives

It should be noted that the CBet employed in this study utilized yeast powder as the coating carrier. Yeast powder contains bioactive compounds, including mannan-oligosaccharides, nucleotides, and β-glucans, which have independently demonstrated regulatory effects on rumen microbiota. For instance, mannan-oligosaccharides can bind rumen pathogens, enhance proliferation of beneficial taxa such as Firmicutes, and promote rumen fermentation to increase volatile fatty acid production [33,60,61]. However, this study did not establish a yeast powder-only control group (with yeast powder supplementation equivalent to that present in the CBet formulation without betaine). This omission limits our ability to differentiate between the independent regulatory effects of betaine’s methyl donor function and the yeast powder carrier’s bioactive constituents on rumen microbiota and subsequent lamb meat quality. Furthermore, this study did not confirm the slow-release characteristics of CBet in the rumen nor quantify the individual contributions of rumen-available betaine, yeast powder carrier, and slow-release mechanisms to rumen fermentation and volatile fatty acid (VFA) production.

Future studies should establish a yeast powder carrier control group to clearly distinguish between the contributions of betaine and yeast powder. The actual rumen-bypass rates should be verified using in vitro rumen incubation or in vivo tracer experiments. Moreover, the slow-release properties of CBet in the rumen should be validated through continuous fermentation experiments in vitro, and gradient addition levels of both CBet and yeast powder should be examined to quantify their independent and combined effects on rumen fermentation. This approach would allow more accurate elucidation of the mechanisms by which CBet improves lamb meat quality and flavor.

Additionally, the relatively small sample size (9 lambs per group) and limited number of replicates (3 replicates) employed in this study could restrict statistical power and generalizability of the findings due to individual variability among lambs. Therefore, future research should prioritize validating these results in larger experimental groups when adequate resources and animal subjects are available, thereby confirming the stability and broader applicability of the current conclusions.

## 5. Conclusions

This study demonstrates that dietary supplementation with 0.20% CBet in weaned lambs modulates the rumen microbial community structure by significantly increasing the Firmicutes-to-Bacteroidota ratio and enriching core functional genera, resulting in substantial elevation of ruminal VFAs. Consequently, CBet supplementation enhances lamb meat physicochemical characteristics (e.g., muscle redness), improves nutritional parameters (e.g., intramuscular fat content), and optimizes volatile flavor profiles by increasing beneficial flavor compounds and reducing undesirable off-flavors. Correlation analysis revealed a clear regulatory cascade from rumen microbiota through fermentation parameters to muscle volatile flavor compounds. Notably, a novel regulatory mechanism was identified whereby Dialister indirectly reduces hexanal accumulation by modulating ruminal butyric acid content, providing new experimental insights into how CBet influences lamb meat quality. In summary, CBet supplementation effectively enhances lamb meat quality and flavor through targeted synergistic regulation of rumen microbiota and muscle metabolism, establishing both theoretical and practical foundations for precise nutritional interventions aimed at optimizing meat quality in ruminants.

## Figures and Tables

**Table 1:** Effects of dietary CBet supplementation on muscle fatty acids (%).

Items	CON	CBet	*p* Value
Saturated fatty acid (SFA)			
Butyric acid, C4:0	0.21 ± 0.05	0.15 ± 0.04	0.398
Caproic acid, C6:0	0.15 ± 0.02	0.18 ± 0.07	0.670
Caprylic acid, C8:0	0.12 ± 0.03	0.18 ± 0.02	0.171
Capric acid, C10:0	0.17 ± 0.04	0.15 ± 0.00	0.787
Lauric acid, C12:0	0.11 ± 0.00	0.11 ± 0.00	0.128
Myristic acid, C14:0	2.11 ± 0.27	2.23 ± 0.14	0.722
Pentadecanoic acid, C15:0	1.33 ± 0.41	1.86 ± 0.25	0.332
Palmitic acid, C16:0	22.69 ± 1.25	20.29 ± 1.32	0.257
Margaric acid, C17:0	1.08 ± 0.10 ^b^	1.39 ± 0.06 ^a^	0.050
Stearic acid, C18:0	13.10 ± 1.31	11.43 ± 0.34	0.285
Arachidic acid, C20:0	0.06 ± 0.00	0.05 ± 0.00	0.940
Heneicosanoic acid, C21:0	0.10 ± 0.01	0.14 ± 0.01	0.084
Behenic acid, C22:0	0.12 ± 0.02	0.15 ± 0.01	0.243
Tricosanoic acid, C23:0	2.96 ± 0.47 ^b^	4.83 ± 0.41 ^a^	0.041
Lignoceric acid, C24:0	0.03 ± 0.00	0.02 ± 0.00	0.367
Monounsaturated fatty acid (MUFA)			
Myristoleic acid, C14:1	0.10 ± 0.01	0.13 ± 0.01	0.128
Palmitic acid, C15:1	0.15 ± 0.01	0.16 ± 0.01	0.531
Palmitoleic acid, C16:1	1.49 ± 0.15	1.73 ± 0.26	0.462
Margaroleic acid, C17:1	0.70 ± 0.09	0.72 ± 0.18	0.905
Trans-9-Elaidic acid, C18:1n9t	4.15 ± 0.26	5.09 ± 0.31	0.084
Cis-9-Elaidic acid, C18:1n9c	32.08 ± 1.53	34.21 ± 1.88	0.429
Cis-11-Eicosenoate acid, C20:1	0.10 ± 0.01	0.11 ± 0.01	0.778
Erucic acid, C22:1n9	0.31 ± 0.08	0.29 ± 0.05	0.864
Nervonic acid, C24:1	0.41 ± 0.07	0.53 ± 0.10	0.376
Polyunsaturated fatty acid (PUFA)			
Trans-Linolelaidic acid, C18:2n6t	0.37 ± 0.11	0.13 ± 0.03	0.093
Cis-Linoleate acid, C18:2n6c	11.08 ± 2. 11	12.95 ± 1.87	0.711
γ-linolenic acid, C18:3n6	0.09 ± 0.01	0.12 ± 0.01	0.102
Arachidonic acid, C20:4n6	0.05 ± 0.01	0.10 ± 0.03	0.193
Docosahexaenoic acid, C22:6n3	0.21 ± 0.04	0.23 ± 0.04	0.789
ɑ-Linolenic acid, C18:3n3	0.25 ± 0.07	0.26 ± 0.02	0.857
Eicosadienoate acid, C20:2	0.11 ± 0.02	0.11 ± 0.03	1.000
Eicosatrienoate acid, C20:3n6	0.19 ± 0.03	0.30 ± 0.04	0.105
Eicosatrienoic acid, C20:3n3	0.07 ± 0.01	0.09 ± 0.02	0.277
Docosadienoic acid, C22:2	0.15 ± 0.02 ^a^	0.07 ± 0.01 ^b^	0.012
Eicosapentaenoic acid, C20:5n3	0.09 ± 0.04	0.11 ± 0.03	0.653
Saturated fatty acid (SFA)	44.22 ± 1.88	43.03 ± 0.77	0.591
Unsaturated fatty acid (UFA)	53.94 ± 2.22	56.22 ± 0.61	0.374
Monounsaturated fatty acid (MUFA)	39.49 ± 1.61	42.95 ± 1.61	0.221
Polyunsaturated fatty acid (PUFA)	13.08 ± 2.73	14.64 ± 0.88	0.614

Values are expressed as means ± SEM (*n* = 6), ^a,b^ Values within the same row bearing different superscripts indicate significant differences (*p*  <  0.05).

**Table 2:** Effects of dietary supplementation with CBet on volatile flavor compounds in muscle (%).

Classification	Compound	CON	CBet	*p* Value
Aldehyde	Hexanal	18.87 ± 3.26	15.78 ± 2.99	0.417
	Heptanal	12.92 ± 4.51	12.33 ± 2.25	0.268
	Octanal	11.96 ± 4.21	12.79 ± 2.70	0.112
	Nonanal	12.80 ± 1.37 ^b^	15.95 ± 2.91 ^a^	0.010
	Benzaldehyde	1.98 ± 0.81 ^b^	2.13 ± 0.83 ^a^	0.010
	Decanal	0.16 ± 0.07	0.25 ± 0.08	0.194
	Dodecanal	0.28 ± 0.06 ^a^	0.06 ± 0.01 ^b^	0.023
	2-Nonenal, (E)	0.30 ± 0.08	0.28 ± 0.09	0.126
	2-Decenal, (E)	1.96 ± 0.28 ^a^	0.58 ± 0.11 ^b^	0.028
	2,4-Nonadienal, (E,E)	2.23 ± 0.61	2.63 ± 0.18	0.464
	2,4-Decadienal, (E,E)	0.18 ± 0.10	0.12 ± 0.07	0.202
	2-Octenal, (E)	0.11 ± 0.06	0.37 ± 0.13	0.209
	3-Methylbutanal	ND	0.27 ± 0.09	
Alcohols	1-Decanol, 2-ethyl-	0.11 ± 0.06	0.12 ± 0.02	0.185
	1-Nonen-3-ol	0.08 ± 0.02	0.03 ± 0.01	0.132
	1-Octen-3-ol	0.33 ± 0.04 ^b^	1.20 ± 0.94 ^a^	0.032
	1-Nonanol	0.23 ± 0.03 ^a^	0.04 ± 0.01 ^b^	0.04
	1-Dodecen-3-ol	0.22 ± 0.10	0.03 ± 0.01	0.149
	Cycloheptanol	2.13 ± 0.98 ^a^	1.02 ± 0.01 ^b^	0.025
	2-Nonen-1-ol	0.03 ± 0.01	0.03 ± 0.01	0.785
	2-Decen-1-ol, (E)	ND	0.02 ± 0.01	
	Cyclodecanol	ND	0.44 ± 0.08	
	6-Methyl-1-heptanol	ND	0.08 ± 0.02	
Hydrocarbon	Toluene	17.05 ± 2.75	19.21 ± 3.76	0.334
	Ethylbenzene	2.04 ± 1.02 ^a^	1.32 ± 0.14 ^b^	0.039
	p-Xylene	2.05 ± 0.19 ^a^	1.28 ± 0.43 ^b^	0.042
	o-Xylene	3.34 ± 1.05 ^a^	2.00 ± 0.42 ^b^	0.021
	1,3-Dimethylbenzene	4.59 ± 1.11	3.96 ± 1.25	0.067
	Ethylidenecycloheptane	ND	0.58 ± 0.22	
	Styrene	0.25 ± 0.11	0.18 ± 0.02	
Ketones	6-Methyl-5-hepten-2-one	0.30 ± 0.15	0.28 ± 0.11	0.534
Heterocyclic	2-Hexylfuran	ND	0.28 ± 0.12	
	(Hexadecyloxy)methyloxirane	ND	0.02 ± 0.01	

Values are expressed as means ± SEM (*n* = 6); ND indicates not detected. ^a,b^ Values within the same row bearing different superscripts indicate significant differences (*p*  <  0.05).

**Table 3:** ROAV of volatile flavor compounds in two groups.

Compound	CAS	Odor Threshold (μg/kg)	ROAV		Odor Description
			CON	CBet	
Hexanal	66-25-1	4.2	19.15	12.05	Grassy, fresh, tallow, fat
Heptanal	111-71-7	2.8	20.88	14.85	Grassy, fresh, tallow, fat
Octanal	124-13-0	0.6	36.83	30.12	Grease, citrus, soapy
Nonanal	124-19-6	0.9	24.89	29.54	Floral, aldehyde-like, citrus, soapy, fried fragrant, roasted fragrant
Benzaldehyde	100-52-7	28.5	0.24	0.24	Almond, burnt sugar, sweet
Decanal	112-31-2	0.9	0.13	0.18	Fresh grease, fruity, soapy
Dodecanal	112-54-9	1.07	0.18	0.03	Orange-like, faint fatty
2-Nonenal, (E)	18829-56-6	0.9	0.23	0.19	Rancid, green, fatty
2-Decenal, (E)	3913-81-3	0.3	23.02	5.33	Green, cucumber-like, fatty
2,4-Nonadienal, (E,E)	5910-87-2	0.05	100	100	Fatty, grassy, roasted meat, nutty
2,4-Decadienal, (E,E)	25152-84-5	0.07	5.83	2.96	Fatty, fried, roasted fragrant
2-Octenal, (E)	2548-87-0	0.35	0.89	2.4	Meaty, nutty, grease, cucumber flavor, umami
3-Methylbutanal	590-86-3	1.8	ND	0.45	Malt-like, roasted, nutty
1-Nonen-3-ol	21964-44-3	18	0.01	0.02	Mushroom, earthy, fungal
1-Octen-3-ol	3391-86-4	1	1.91	8.81	Earth, fat, floral, green, herb
Cycloheptanol	502-41-0	1000	0.07	0.03	Faint alcoholic, woody
Cyclodecanol	1502-05-2	1000	ND	0.01	Faint woody, fatty
Toluene	108-88-3	3500	0.17	0.15	Bitter almond, glue, paint
Ethylbenzene	100-41-4	2000	0.03	0.02	Faint aromatic, solvent-like
p-Xylene	106-42-3	2250	0.03	0.02	Faint aromatic, sweet
o-Xylene	95-47-6	1500	0.08	0.04	Faint aromatic, solvent-like
Benzene, 1,3-Dimethylbenzene	108-38-3	1850	0.09	0.08	Faint aromatic, sweet
6-Methyl-5-hepten-2-one	110-93-0	6	0.18	0.16	Fruity, lemon grass, citrus
2-Hexylfuran	3777-70-6	6	ND	0.18	Floral, fruit, green, green bean

ND means not detected.

## Data Availability

The data supporting the findings of this study are available from the corresponding author upon reasonable request.

## Supplementary Materials

The following supporting information can be downloaded at: https://www.mdpi.com/article/10.3390/ani16060970/s1, Table S1: Basic diet formulation; Table S2: Alpha diversity index analysis.
