# Supplementary material for: Coated Betaine Improves Lamb Meat Quality and Flavor by Modulating Rumen Microbial Flora
Source: Animals (Basel). 2026 Mar 20;16(6):970. doi: 10.3390/ani16060970 (PMC13023291; doi:10.3390/ani16060970)
Supplement: Supplementary file 1 [file animals-16-00970-s001.zip › animals-4171328-supplementary.pdf]

**Table S1** Basic diet formulation

| Items | Diets (%) | Nutrient levels | |
|---|---|---|---|
| Corn | 32.0 | digestive energy DE/（MJ/kg） | 11.02 |
| Wheat bran | 6.0 | Moisture/% | 11.76 |
| Grass meal | 10.0 | CP/% | 16.43 |
| Wheatgrass | 10.0 | Ash/% | 8.16 |
| Soybean meal（43%） | 5.0 | salinity/% | 0.96 |
| Cottonseed meal（46%） | 5.0 | Ca/% | 1.23 |
| Bran | 8.0 | | |
| Corn germ meal | 15.0 | | |
| Alcohol grains | 5.0 | | |
| Premix[a] | 4.0 | | |

[a] **Composition (per kg of dry matter):** 100,000–500,000 IU of vitamin A, 50,000–200,000 IU of vitamin $D_3$, ≥500 IU of vitamin E, Fe 1,500-7,000 mg, Cu 300-750 mg, Mn 1,000-5,000 mg, Zn 1,500-4,000 mg, I 20-30 mg, Se 5-20 mg, Co 8-35 mg.

**Table S2** Alpha diversity index analysis

| Items | CON | CBet | *P*-value |
|---|---|---|---|
| Chao1 index | 668.95±24.36 | 749.49±44.10 | 0.149 |
| Ace index | 676.54±24.85 | 758.72±43.37 | 0.139 |
| Shannon index | 6.47±0.08 | 6.55±0.17 | 0.614 |
| Simpson index | 0.97±0.01 | 0.97±0.00 | 0.913 |

Values are expressed as means±SEM (n=6). [a,b]The values within a row with different superscripts are significantly different ($P < 0.05$).
